# Effect of Equal Volume Replacement of Fine Aggregate with Fly Ash on Carbonation Resistance of Concrete

**DOI:** 10.3390/ma15041550

**Published:** 2022-02-18

**Authors:** Dongsheng Zhang, Yafan Wang, Mingxiao Ma, Xiangjun Guo, Shuangquan Zhao, Shuxiang Zhang, Qiuning Yang

**Affiliations:** 1 School of Civil and Hydraulic Engineering, Ningxia University, Yinchuan 750021, China; dongsheng.zhang@kuleuven.be (D.Z.); wyf990710@163.com (Y.W.); mamingxiao2022@163.com (M.M.); zsxiang4531@163.com (S.Z.); 2 Research Group RecyCon, Department of Civil Engineering, KU Leuven, Campus Bruges, 8200 Bruges, Belgium; 3 China Construction Third Engineering Bureau Group Co., Ltd., Wuhan 430223, China; xiangjunguo2012@163.com (X.G.); shuangquanzhao2011@163.com (S.Z.)

**Keywords:** concrete with fly ash as fine aggregate, carbonation depth, pH, pore structure

## Abstract

Concrete is prepared by substituting an equal volume of fly ash for fine aggregate, and the effect of substitution rate on its carbonation resistance is studied. Using a rapid carbonation test, the distribution law of the internal pH value of concrete with fly ash as fine aggregate (CFA) along the carbonation depth under different substitution rates (10%, 20%, 30%, and 40%) after carbonation is studied and compared with the test results of phenolphthalein solution. Then, to further clarify the damage mechanism of fly ash replacing fine aggregate on concrete carbonation, the changes in the pore structure and micromorphology of CFA after carbonation are studied by means of mercury intrusion pressure and electron microscope scanning tests. The results indicate that the carbonation depth of CFA increases gradually with increasing carbonation time. In particular, in the later stage of carbonation, the carbonation rate of concrete decreases significantly with an increase in the substitution rate. The carbonation depth X_C_ of CFA measured by phenolphthalein solution is approximately 0.24–0.39 times of the complete noncarbonation depth measured by the pH method. The pH value test is a reliable test method that can reveal the carbonation mechanism of CFA. Carbonation can significantly reduce the proportion of more harmful holes in concrete with a large amount of fly ash, but it can also increase the proportion of less harmful and harmful holes. In general, the pore size distribution and micromorphology of concrete can be improved by replacing fine aggregates with fly ash.

## 1. Introduction

Fine aggregate is an indispensable component of concrete [1,2]. Currently, fine aggregate in concrete is mainly natural river sand; however, natural river sand is a nonrenewable resource that has been formed over hundreds of millions of years. With the continuous and rapid development of infrastructure, the amount of concrete is huge, and the demand for natural river sand is increasing daily. In most parts of China and even globally, there has been a shortage of natural river sand resources or even no natural river sand [3]. Therefore, there is an urgent need to determine substitutes for river sand. Thus, reasonable and effective recycling of fly ash has significant social, environmental, and economic benefits. A new type of green concrete with fly ash as fine aggregate (CFA) is developed [4,5,6,7,8]. Fly ash is an industrial waste product. Using its microaggregate effect to replace fine aggregate not only breaks through the limitation of the conventional utilisation of fly ash in the concrete field and improves its use value, but also can alleviate the current situation of the shortage of natural river sand resources and reduce pollution.

Concrete durability is an important factor affecting the service life of concrete structures [9,10,11]. It is generally believed that the addition of fly ash (instead of cement) can improve the durability of concrete by reducing its permeability. However, carbonation is one of the main factors affecting the durability of concrete [12,13,14], and the influence of replacing fine aggregates with fly ash on concrete is not fully understood; therefore, it is necessary to study the carbonation resistance of CFA.

Carbonation occurs when acidic gases such as CO_2_ form carbonic acid in the concrete, and react with alkaline substances such as the cement hydration product Ca(OH)_2_ to form CaCO_3_, which reduces the alkalinity of concrete [15,16,17]. Currently, carbonation becomes more severe with an increase in CO_2_ concentration in the environment [18]. However, higher requirements have been proposed for the anticarbonation performance of concrete. Carbonation depth is an important parameter adopted to the carbonation resistance of concrete. The standard measurement method for concrete carbonation depth is the phenolphthalein test method, which is widely employed worldwide [19,20]. When the critical pH value of the phenolphthalein solution in concrete is approximately 9.0 [21,22], the carbonation depth of the concrete is inferred. Some prediction models of concrete carbonation depth based on CO_2_ diffusion in porous media are also deduced based on the experimental data obtained using this method.

In fact, some studies and field tests have indicated that when the alkalinity of concrete is lower than 11.5, the reinforcement begins to de-blunt and rust [23]. Only the depth of the complete carbonation zone measured by the phenolphthalein solution exists, and there is still a partial carbonation zone inside the concrete [24,25], in which Ca(OH)_2_ and CaCO_3_ coexist. Obviously, CO_2_ gas is diffused into the concrete with a deeper carbonation depth measured by phenolphthalein, reducing the internal pH value. Therefore, in addition to the complete carbonation area, the reinforcement in the partial carbonation area of concrete may be corroded when the pH is greater than 9.0. The phenolphthalein test method leads to an underestimation of the carbonation depth of concrete [24]. The time at which the reinforcement corrosion begins to de-blunt, calculated according to the prediction model, may be inconsistent with the actual situation. At the same time, information obtained by this method is too small to understand the carbonation process inside concrete. Therefore, the pH value of the simulated pore solution has been used by some scholars to characterise the alkalinity change in concrete pore solutions caused by carbonation.

The preparation of concrete based on fly ash instead of fine aggregate can not only recover a large amount of waste discharged from thermal power plants, but also save nonrenewable resources. To clarify the influence of fly ash replacing fine aggregate on the carbonation performance of concrete, the carbonation depth of CFA is measured using the phenolphthalein method and a pH value test, and the carbonation depth values are compared and analysed. At the same time, the changes in the pore structure and micromorphology of CFA after carbonation are studied by means of mercury intrusion pressure and electron microscope scanning tests to further reveal the mechanism of action of fly ash replacing fine aggregate in concrete carbonation.

## 2. Experiment Details

### 2.1. Raw Material

Ordinary Portland cement (PO 42.5) and grade III fly ash were used in this study, and their physical and chemical properties are respectively presented in Table 1 and Table 2. The fine aggregate was natural river sand, the fineness modulus was 2.76, the mud content was 2.5%, the bulk density was 1490 kg/m^3^, the apparent density was 2660 kg/m^3^, and the coarse aggregate was natural gravel with a particle size of 5–20 mm, mud content of 0.5%, crushing index of 9.1%, and apparent density of 2720 kg/m^3^. Superplasticiser is a polycarboxylic acid high-performance superplasticiser with a solid content of 30%, a water reduction rate of 29.7%, and ordinary tap water.

### 2.2. Mix Design and Preparation

Using ordinary concrete with a water–cement ratio of 0.42 as the reference group (OPC), under the same water–cement ratio, CFA is composed of fly ash equivalent volume instead of fine aggregate [26,27]. The substitution rates of fly ash are 10%, 20%, 30%, and 40%, respectively, which are CFA-10, CFA-20, CFA-30, and CFA-40, respectively. Equation (1) is the calculation formula for the unit consumption of fly ash in concrete:

(1) $$M_{FA}=\frac{M_{S}\times R\times \rho_{FA}}{\rho_{S}}$$

where $\rho_{S}$ and $\rho_{FA}$ are the densities of fine aggregate and fly ash, respectively; $M_{S}$ and $M_{FA}$ are the unit dosages of fine aggregate and fly ash, respectively; and *R* is the substitution rate of fly ash. The concrete mix proportions are listed in Table 3.

### 2.3. Carbonation Test Design

**Figure 1:**
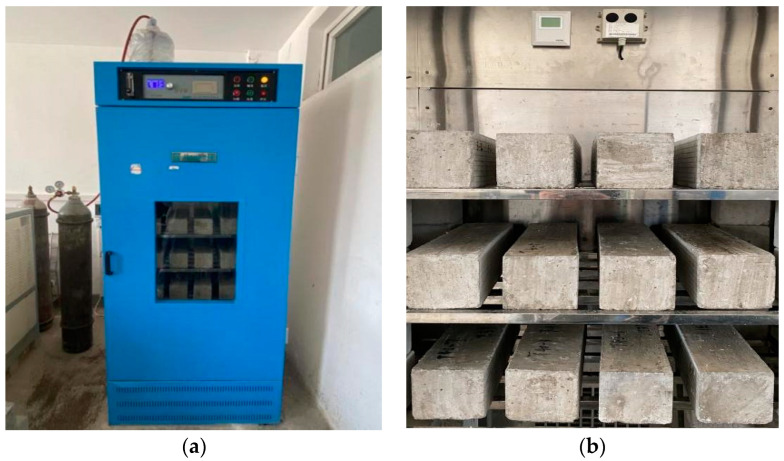
Rapid carbonation box. (**a**) Exterior; (**b**) interior.

**Figure 2:**
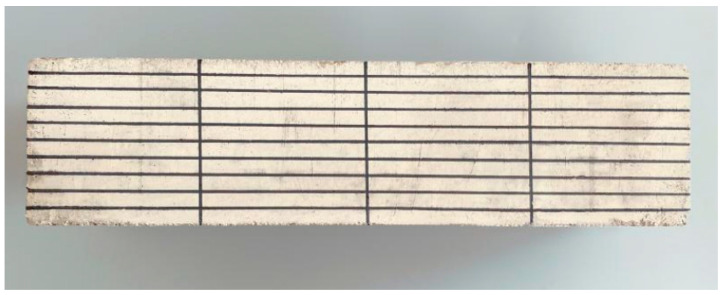
Specimen to be measured.

According to the Chinese standard GB/T 50082-2009 [19], the concrete moulding is 100 mm × 100 mm × 400 mm prisms, and three test pieces are made for each mix proportion for the determination of carbonation depth by the phenolphthalein solution method and the determination of the pH value of the apparent pore solution, MIP, and SEM. After the specimen is formed for 24 h, the mould is removed, and the specimen is placed in a standard curing room for curing. After curing for 26 d, the specimen is placed in a 60 °C oven and dried for 48 h. After removing it, apart from leaving two opposite nonformed sides, the other surfaces are sealed with heated paraffin. The specimens are then placed into a carbonation test chamber (Figure 1) at a temperature of 20 ± 2 °C, humidity of 70 ± 5%, and CO_2_ concentration of 20 ± 3% to accelerate carbonation to 7, 14, 28, and 56 d. The specimens are taken out and sampled by the splitting method (Figure 2).

### 2.4. Test Method for Carbonation Depth

#### 2.4.1. Phenolphthalein Reagent Method

**Figure 3:**
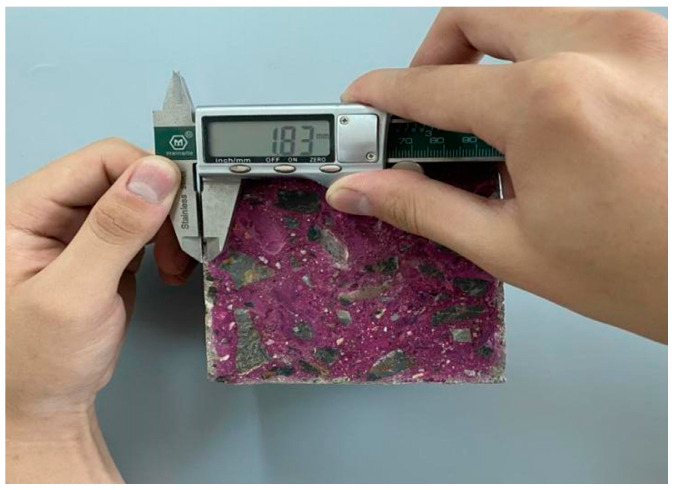
Carbonation depth measurement.

When carbonation reaches 7, 14, 28, and 56 d, the test pieces of each proportion are removed, the shape is broken from one end by the splitting method, the powder is scraped off on the cut section, and then the phenolphthalein indicator is sprayed at a concentration of 1%. The depth of the complete carbonation zone can be determined according to the colour change of the indicator. After 30 s, the carbonation depth of each measuring point is measured using a digital Vernier calliper, and the arithmetic average of each measuring point is considered as the carbonation depth of the test piece (as illustrated in Figure 3). The cutting surface of the remaining specimen is sealed with paraffin and then placed into a carbonation box to continue carbonation until the next test age.

#### 2.4.2. Determination of Apparent pH Value of Concrete Pore Solution/Determination of pH Value of Concrete Pore Solution

**Figure 4:**
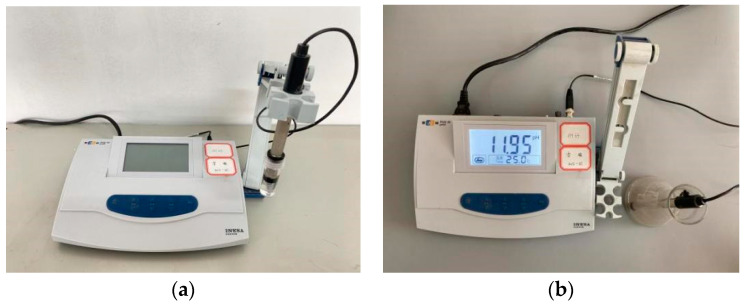
pH measurement. (**a**) Model PHS-3E pH instrument; (**b**) measuring processes.

A microbench drill was used to sample the drill powder of the test block along the carbonation depth direction, with one sample every 2 mm, and the sampling depth was 2 cm. After grinding and sieving the sample, the sample was placed in a 105 °C oven to dry for 24 h; then 10 g of each sample was weighed and dissolved in 100 mL distilled water, plugged with a rubber plug to prevent further carbonation, stirred with a magnetic stirrer for 30 min, and then shaken with an oscillator for 1 h. After 72 h, the pH of the solution was measured using a PHS-3E pH meter, as shown in Figure 4.

### 2.5. Pore Structure Analysis

A 5 mm sample of the specimen was cut, and the coarse aggregate was removed and placed in absolute ethanol to stop hydration, and the sample bottle with paraffin was sealed to prevent volatilisation and dried in a 60 °C oven before testing [28]. An AutoPore IV 9500 mercury injection instrument (MIP) was used, and the pore size distribution of the sample was calculated using the Washburn equation. The variation in macrophenomena, such as the carbonation depth in the carbonation process, can be fully explained by mercury injection experimental data.

### 2.6. SEM

The test sample selection was the same as that used in the mercury injection test. After sampling, the samples were soaked in absolute ethanol to stop cement hydration. Before the test, the sample was dried in an oven at 60 °C until the quality remained unchanged. The micromorphology of the samples was observed using a Quanta 400 FEG scanning electron microscope (SEM). To ensure a clear image, gold spray was used.

## 3. Results and Analysis

### 3.1. Carbonation Depth Test

#### 3.1.1. Analysis of Phenolphthalein Solution Test Results

**Figure 5:**
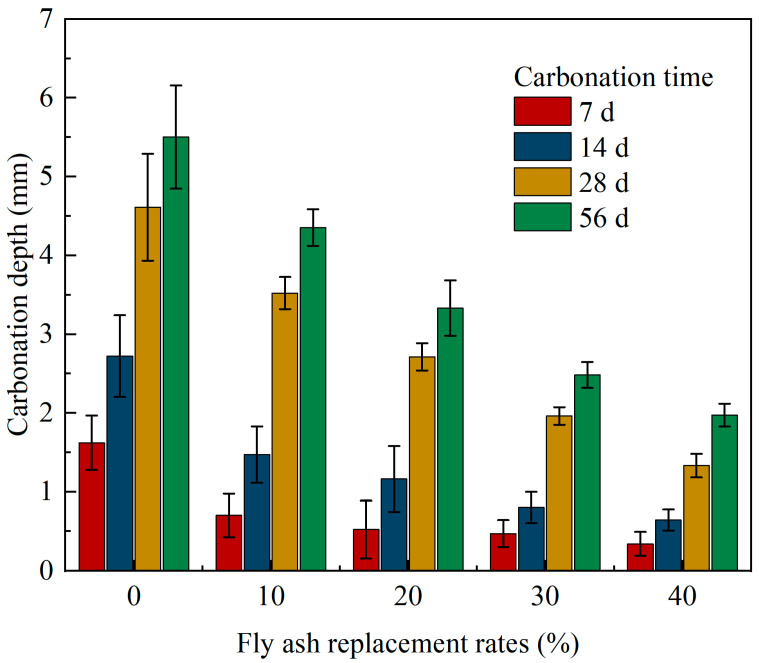
Carbonation depth changes of concrete with different fly ash replacement rates at different carbonation ages.

The change in the carbonation depth of concrete with different fly ash replacement rates under each carbonation age is shown in Figure 5. It can be observed from Figure 5 that the carbonation depth of each specimen gradually increased with the increase in carbonation time. However, the carbonation rate of concrete decreases obviously in the last stage of carbonation, as illustrated in Table 4, when carbonised to 56 days. The carbonation rates of CFA with replacement rates of 0%, 10%, 20%, 30%, and 40% are 0.09, 0.08, 0.06, 0.04, and 0.04 mm/d, which are only 0.39, 0.80, 0.86, 0.57, and 0.80 times the carbonation rate of 7 days, respectively. This is similar to the previous concrete carbonation test results and theoretical results. The reason for this phenomenon is that the CO_2_ concentration gradient decreases with an increase in the carbonation depth; however, CaCO_3_ generated in the carbonation process fills part of the pores of concrete, making the concrete structure denser, thus reducing the air permeability of concrete and hindering the further diffusion of CO_2_, which reduces the carbonation rate [29,30,31].

For fly ash instead of fine aggregate, the carbonation depth of concrete decreases with an increase in the substitution rate. When carbonised to 56 days, the carbonation depth of CFA with replacement ratios of 10%, 20%, 30%, and 40% is 4.35, 3.33, 2.48, and 1.97 mm, respectively, which is only 79.09%, 60.55%, 45.09%, and 35.82% that of ordinary concrete. This is different from the previous concrete with fly ash instead of cement [32,33], and its carbonation depth often increases with an increase in the fly ash content; for fly ash concrete, the addition of fly ash reduces the amount of cement. Therefore, the content of carbonised materials such as Ca(OH)_2_ and C-S-H gel can be reduced, and the content of Ca(OH)_2_ is further reduced by the secondary hydration reaction [34,35]; thus, the ability of concrete to resist carbonation decreases. After replacing fine aggregate with fly ash, the amount of cement does not decrease in concrete, although the secondary hydration of fly ash reduces the content of carbonatable substances in CFA; however, fly ash exerts its microaggregate effect uniformly in the voids of aggregate particles [36,37]. The two hydration reactions of fly ash produce C-S-H and C-A-H and other gel products filled into the pores of the interfacial transition zone, which improves the density of concrete and interstices of cement particles, making the concrete denser and reducing the diffusion rate of CO_2_. The inhibition effect of this effect is greater than the promotion effect of cement reduction on carbonation; thus, it comprehensively reduces the carbonation depth of CFA [12].

#### 3.1.2. Analysis of Apparent pH Value of Concrete Pore Solution

**Figure 6:**
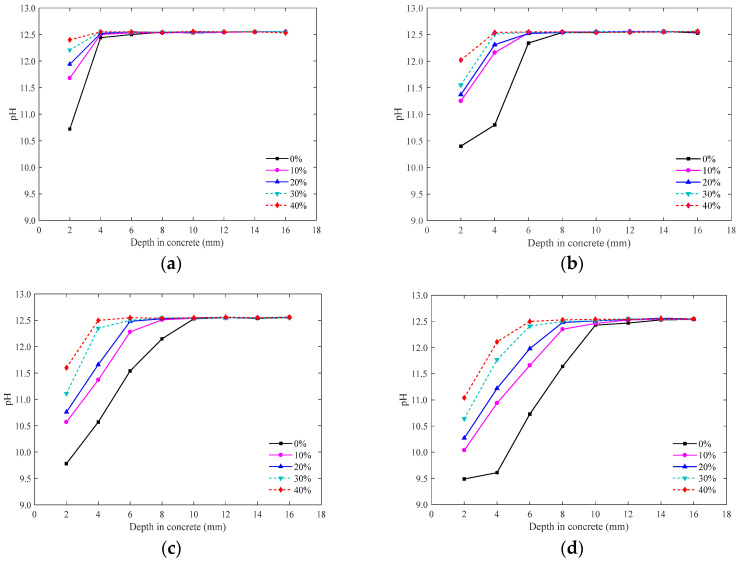
pH value of CFA after carbonation. (**a**) Carbonation-7d, (**b**) carbonation-14d, (**c**) carbonation-28d, (**d**) carbonation-56d.

Figure 6 shows the variation law of the pH value of the pore solution at different depths from the surface of the concrete when the CFA was carbonised for 7, 14, 28, and 56 d under different fly ash substitution rates. As shown in Figure 6, under different substitution rates, the pH value of the concrete surface is much lower than that of the concrete interior because carbonation starts from the concrete surface. With an increase in depth, the decrease in the pH value gradually slows down. This indicates that deeper into the concrete, the amount of CO_2_ diffused to this point is lower, the carbonation degree of concrete is lower, and the pH value remains constant to a certain extent [32]. From Figure 6, the pH value of concrete on the surface of the specimen decreased most obviously, whereas the pH value of concrete at the depth of the specimen remained basically unchanged, which was maintained at approximately 12.55. Compared with early carbonation (7, 14 d), the pH value of the concrete surface continues to decline in the late carbonation (28, 56 d), which is due to the diffusion of CO_2_ into deeper concrete, the carbonation reaction with Ca(OH)_2_ in concrete, and the continuous formation of CaCO_3_.

For fly ash replacing fine aggregate, with the extension of the carbonation age, the pH value of concrete under different fly ash replacement rates shows a downward trend. When carbonated for 56 d, the pH value of concrete 10 mm from the surface was less than 12.55. Compared with ordinary concrete, the pH value of CFA with replacement rates of 10%, 20%, 30%, and 40% decreased slowly, and the pH value at the same depth (10 mm) increased by 0.03, 0.08, 0.08, and 0.11, respectively. Therefore, fly ash can replace fine aggregates to obtain concrete with a superior carbonation resistance.

#### 3.1.3. Comparative Analysis of Phenolphthalein Test Results and pH Value Test Results

As a traditional test method, phenolphthalein solution is simple to operate; however, it cannot reflect the change law of the alkalinity of concrete along the carbonation depth direction. The method of pH value testing concrete alkalinity has high accuracy, but the test is complex and should not be applied to practical engineering detection [38]. Generally, concrete at different carbonation depths can be divided into three parts: complete carbonation area, incomplete carbonation area, and partial carbonation area. The carbonation depth measured by the phenolphthalein solution is usually regarded as the complete carbonation depth, which is calculated as Xc, whereas the depth with an extremely small pH value change, that is, the boundary value of the partial carbonation area and complete noncarbonation area, is calculated as Xc’ (powder is taken in a 2 mm layer this time, and the error of this value is 2 mm), and the difference between Xc and Xc’ is defined as the partial carbonation depth, which is calculated as Xp [39]. Table 5 presents a comparison between the phenolphthalein test results and the pH test results of CFA after carbonation for 56 d. The results show that:

(1) The carbonation depth Xc measured by phenolphthalein solution is approximately 0.24–0.39 times the complete noncarbonation depth measured by the pH method (mean value is 0.32). According to this result, the influence of carbonation on CFA can be roughly inferred from the carbonation depth obtained by the phenolphthalein test using the pH value test method in this study.
(2) The depth Xp of the partial carbonation zone is approximately 1.55–3.06 times that of the complete carbonation depth X_C_. It can be observed that the carbonation depth of CFA is underestimated by the phenolphthalein solution test method, and the calculated reinforcement plus the passivation time may be inconsistent with reality. Therefore, in the actual design of the carbonation resistance of reinforced CFA, it is more scientific to suggest that the depth of the partial carbonation area should not exceed the thickness of the protective layer of CFA.

### 3.2. Pore Structure Analysis

#### 3.2.1. Porosity and Pore Connectivity

**Figure 7:**
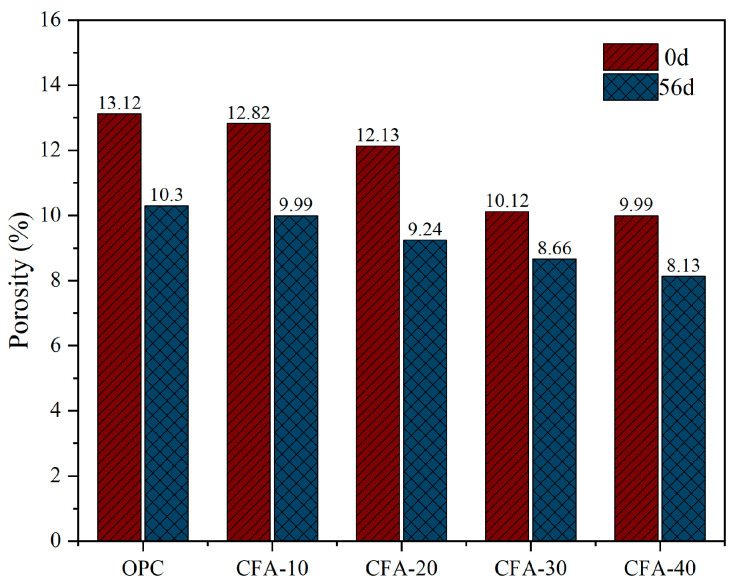
Porosity of concrete before and after carbonation under different replacement rates of fly ash.

Porosity refers to the proportion of void volume to total volume, which has an important impact on the strength and durability of concrete [40]. This can characterise the influence of the fly ash substitution rate on the percentage of cement stone void per unit volume. The porosity of concrete before and after carbonation with different substitution rates of fly ash measured by mercury injection is presented in the Figure 7. Compared with ordinary concrete, the decrease in porosity of CFA10 and CFA20 before the carbonation reaction is not obvious, and the ranges are 2.29% and 7.54%, respectively. The porosities of CFA30 and CFA40 decrease by 22.86% and 23.86%, respectively. For carbonated concrete, the porosity of each group of CFA decreases with an increase in the substitution rate, ranging from 14.4% to 23.8%. The porosity of CFA40 after carbonation is the lowest (8.13%). This also indicates that the replacement of fine aggregate with fly ash can effectively reduce the porosity of concrete, which may be the insoluble product CaCO_3_ generated by the carbonation reaction that fills the pores and reduces the porosity [26]. In addition, owing to the microaggregate effect of fly ash under the secondary hydration of fly ash [41,42], a higher fly ash content can better fill the aggregate gap, thereby improving the compactness of concrete.

However, porosity is not the most important factor affecting permeability. Concrete with high total porosity does not have high permeability [43]. For example, the porosity of CFA20 before and after carbonation is lower than that of CFA10, while the critical pore diameter of CFA20 before and after carbonation is 20.33 and 95.46 nm, respectively, but both are higher than that of CFA10. It can also be observed from the table that although the carbonation reaction causes a decrease in the total porosity of concrete, it increases the critical pore size of each group of samples to varying degrees, improves the connectivity of pores, and makes it easier for harmful ions in the external environment to enter concrete.

#### 3.2.2. Pore Size Distribution

**Figure 8:**
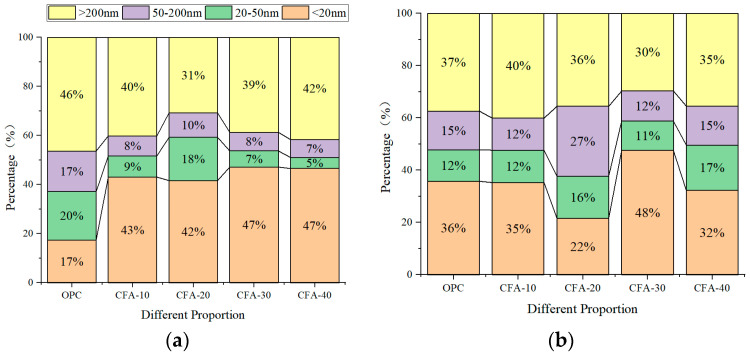
Ratio of pore diameter before and after carbonation of concrete under different fly ash replacement rates. (**a**) Carbonation-0d; (**b**) carbonation-56d.

**Figure 9:**
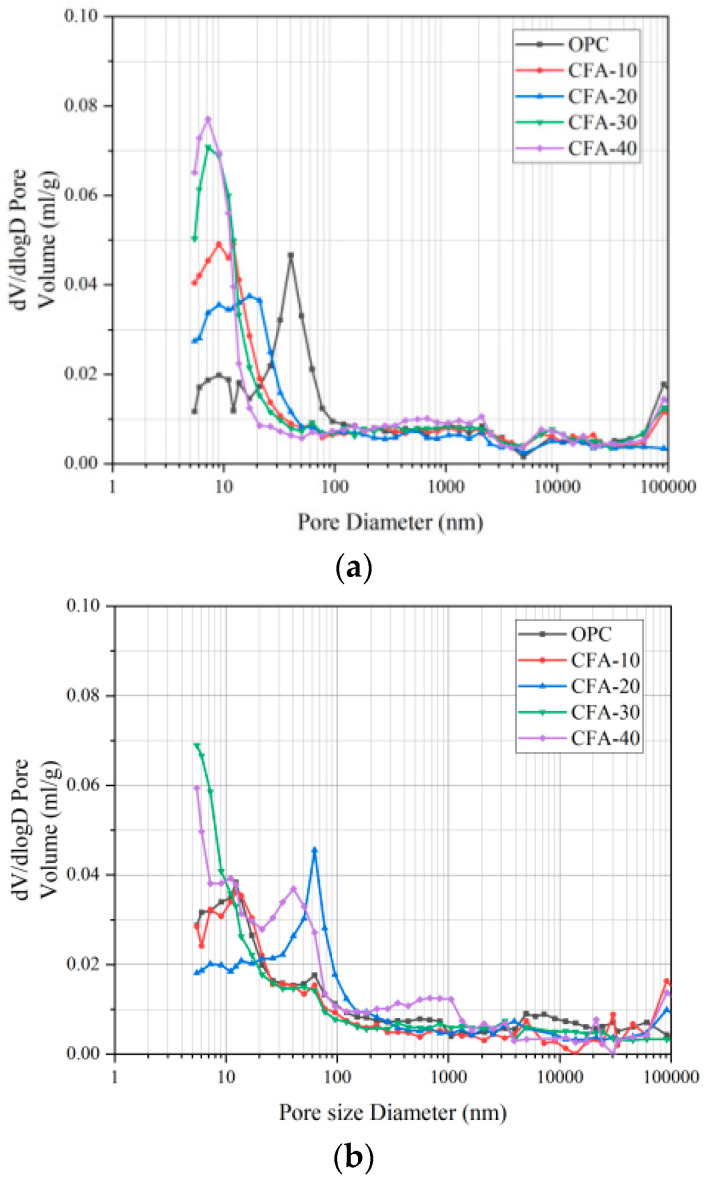
Differential logarithmic curve of mercury intake before and after carbonation of concrete under different fly ash replacement rates. (**a**) Carbonation-0d; (**b**) carbonation-56d.

The pore size distribution of concrete before and after carbonation under different fly ash substitution rates is illustrated in Figure 8 and Figure 9. According to Wu Zhongwei’s pore size division method, the pore grade is divided into four categories: harmless (<20 nm), less harmful (20–50 nm), harmful (50–200 nm), and more harmful (>200 nm) [44,45]. 

As illustrated in Figure 8a, the proportion of harmless pores (<20 nm) in the OPC group before carbonation is only 17%, whereas the proportion of multiharmful pores (>200 nm) is as high as 46%. When fly ash replaces fine aggregates, the number of harmful pores decreases, but this is not significant. This is determined by the particle size gradation of fly ash, which cannot fill the large pores between the skeletons. However, the proportion of holes < 20 nm increases significantly, and the harmless holes in concrete with replacement rates of 10%, 20%, 30%, and 40% are 43%, 42%, 47%, and 47%, respectively. This is because fly ash is filled with fine particles in the cement paste, and pores are refined. The C-S-H gel produced by the hydration of fly ash is approximately insoluble in water, filling the interface of the particles and making the structure denser [46,47]. After 56 days of carbonation, the influence of the pore size distribution of CFA under different substitution rates does not change uniformly. After carbonation, the proportion of harmless holes in ordinary concrete increases, whereas the proportion of less harmful holes, harmful holes, and more harmful holes decreases. The pore size distribution of CFA10 is similar to that of ordinary concrete, and the change is not significant. For CFA20, the proportion of harmless pores decreases significantly from 42% before carbonation to 22%, and the proportion of harmful pores and multiharmful pores increases. For CFA30 and CFA40, the proportion of less harmful and harmful holes increases, but the proportion of more harmful holes decreases. For CFA, carbonation will significantly reduce the proportion of more harmful holes in CFA with a large content, but will also increase the proportion of less harmful and harmful holes.

In addition, according to Figure 9a, the pore diameter of each group of concrete before carbonation has a single peak distribution, and the pores are mostly harmless and less harmful pores. The addition of fly ash shifts the peak position in the direction of the small pore diameter. Overall, replacing fine aggregates with fly ash can improve the pore size distribution in concrete. The optimum substitution rate of fly ash in this study is 40%.

### 3.3. SEM Analysis

**Figure 10:**
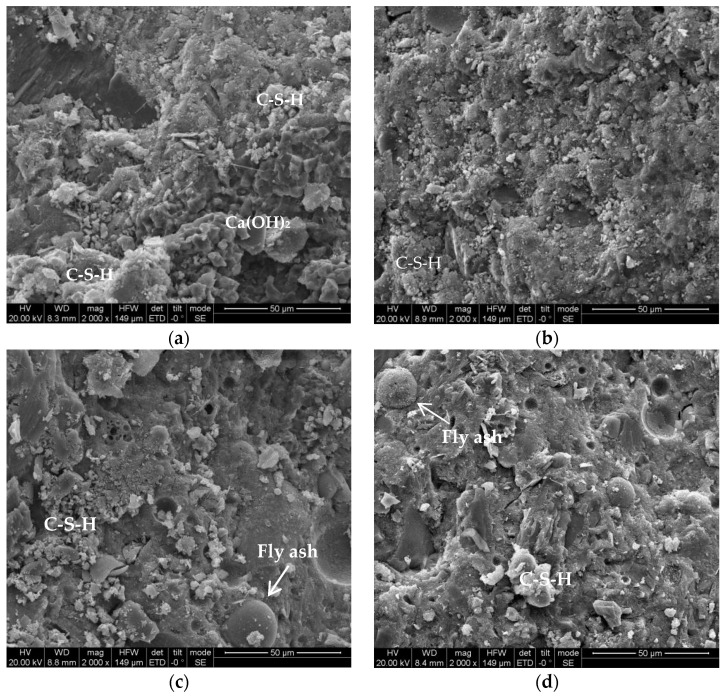

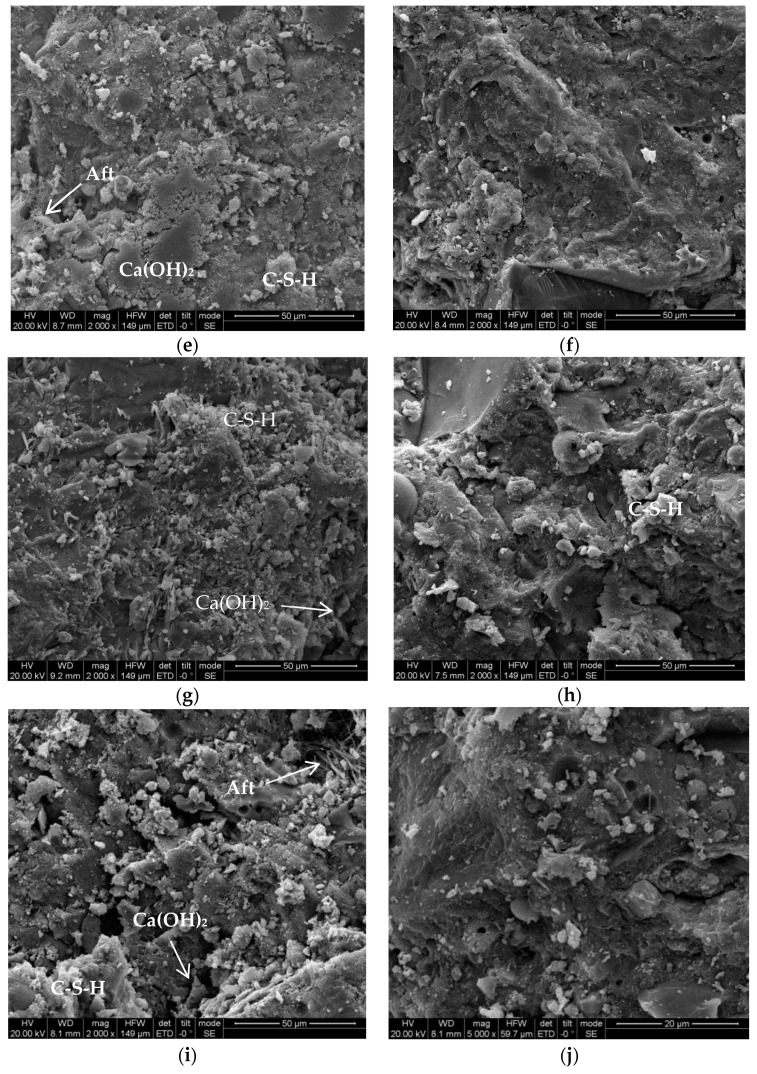
SEM microstructure of concrete before and after carbonation. (**a**) OPC-0d, (**b**) OPC-56d, (**c**) CFA-10-0d, (**d**) CFA-10-56d, (**e**) CFA-20-0d, (**f**) CFA-20-56d, (**g**) CFA-30-0d, (**h**) CFA-30-56d, (**i**) CFA-40-0d, (**j**) CFA-40-56d.

Figure 10 shows a microscopic view of the hydration products of the cement paste samples under accelerated carbonation conditions obtained by electron microscope scanning. There are many pores in concrete, and the microstructure of the interface transition zone between the aggregate and cement paste is related to the compactness of the aggregate [48]. Figure 10a,c,e,g,i shows OPC, CFA-10, CFA-20, CFA-30, and CFA-40 when not carbonised. It can be observed that there are a lot of vermicular C-S-H gel and AFt, some are partially unhydrated fly ash particles in concrete, incomplete crystallisation of Ca(OH)_2_ forms lamellar precipitation in mature cement paste, and the structural density is improved [49]. This is because with the increase in the replacement rate of fly ash, the aggregate fineness is higher and pore filling is denser [47]. Additionally, the C-S-H gel produced by hydration fills the pores in the interfacial transition zone and interacts to strengthen the internal bond strength and increase the integrity [46]. As shown in Figure 10c, only a small number of poorly consolidated block gels were found in the group CFA-10, but no other groups were found. Figure 10b,d,f,h,j shows SEM photos of OPC, CFA-10, CFA-20, CFA-30, and CFA-40 carbonised for 56 days, respectively. Comparing the SEM images of each group before and after carbonation, it was found that the structure of a dense C-S-H gel before carbonisation is gone. Instead of an irregular cluster C-S-H gel structure, a large amount of CaCO_3_ accumulated in the pores and microcracks after carbonation. The number of macropores was significantly reduced compared with that before carbonation, and the number and density of micropores increased. It was also found that CaCO_3_ precipitation in the pores decreased gradually from the OPC to the CFA-40 group. The reasons for these phenomena include an increase in the fly ash substitution rate; the internal structure is gradually dense, which inhibits the infiltration and diffusion of CO_2_ in concrete and reduces the consumption of Ca(OH)_2_; and then it can be inferred that the carbonation degree is also reduced [26]. Therefore, with an increase in the fly ash replacement rate, the carbonation resistance of CFA is continuously enhanced.

## 4. Conclusions

(1) The carbonation depth of CFA increased gradually with increasing carbonation time. In particular, in the later stage of carbonation, the carbonation rate of concrete decreased significantly with an increase in the substitution rate. When carbonated to 56 d, the carbonation rates of CFA with substitution rates of 0%, 10%, 20%, 30%, and 40% were 0.09, 0.08, 0.06, 0.04, and 0.04 mm/d, respectively, which are only 0.39, 0.80, 0.86, 0.57, and 0.80 of the carbonation rate of 7 days.
(2) In contrast to previous concrete with fly ash replacing cement, fly ash replacing fine aggregate significantly improved the carbonation resistance of concrete, and carbonation depth decreased with the increase in fly ash content. This is because the amount of cement was not reduced. Although the secondary hydration of fly ash reduced the content of carbonisable substances in concrete, fly ash contributed to its microaggregate effect and evenly filled the voids of aggregate particles, which improved the compactness of concrete and hindered the diffusion speed of CO_2_. The inhibitory effect was greater than the promotional effect.
(3) In this study, the carbonation depth X_C_ measured by phenolphthalein solution was approximately 0.24–0.39 times that measured by the pH method. The pH value test method is a reliable test method that can reveal the carbonation mechanism of concrete. The distribution characteristics of the apparent pH value of the pore solution in carbonated concrete reflected the carbonation resistance of concrete. From the surface to the interior, the pH of the concrete test block increased and stabilised at a certain value. With an increase in the fly ash content, the pH value of the concrete pore liquid gradually increased, the pH value of the surface concrete pore liquid increased most significantly, and the increased range of the pH value gradually decreased with an increase in the test depth.
(4) Replacing fine aggregate with fly ash effectively reduced the porosity of concrete, which was the insoluble product CaCO_3_ generated by the carbonation reaction that filled the pores and reduced the porosity. In addition, owing to the microaggregate effect of fly ash under the secondary hydration of fly ash, a higher fly ash content filled the aggregate gap, thereby improving the compactness of concrete. Although the carbonation reaction reduced the total porosity of concrete, it increased the critical pore size and improved its pore connectivity.
(5) Carbonation significantly reduced the proportion of more harmful holes in concrete with a large amount of fly ash fine aggregate, but also increased the proportion of less harmful holes and harmful holes. With an increase in the fly ash substitution rate, the number of pores and microcracks on the surface of the concrete matrix gradually decreased. In general, the pore size distribution and the micromorphology of concrete were improved by replacing fine aggregates with fly ash.

## Figures and Tables

**Table 1:** Physical properties of cement and fly ash.

Properties	Cement	Fly Ash
Density (g/cm^3^)	3.04	2.13
Water demanded (%)	28	97
Loss on ignition (%)	3.42	6.3
Fineness (% retained in 45 μm)	5.9	37.1

**Table 2:** Mix proportions of concrete (kg/m^3^).

	SiO_2_	Al_2_O_3_	CaO	Fe_2_O_3_	MgO	SO_3_	P_2_O_5_	Na_2_O	K_2_O
Cement	22.8	7.5	56.1	3.69	0.036	4.38	0.052	0.8	1.04
Fly ash	22.46	7.60	57.15	5.00	1.54	2.96	0.105	0.31	0.86

**Table 3:** Mix proportions of concrete (kg/m^3^).

Group	Water	Cement	Sand	Fly Ash	Gravel	Superplasticiser (%)
OPC	160	381	797	0	1277.2	1.9
CFA-10	160	381	717.3	63.82	1277.2	2.0
CFA-20	160	381	637.6	127.64	1277.2	2.4
CFA-30	160	381	557.9	191.46	1277.2	2.8
CFA-40	160	381	478.2	255.28	1277.2	3.2

**Table 4:** Carbonation rate of concrete with different fly ash replacement rates at different carbonation ages.

Carbonation Rate (mm/d)	7 d	14 d	28 d	56 d
OPC	0.23	0.19	0.16	0.09
CFA-10	0.10	0.11	0.13	0.08
CFA-20	0.07	0.08	0.10	0.06
CFA-30	0.07	0.06	0.07	0.04
CFA-40	0.05	0.05	0.05	0.04

**Table 5:** Results comparison of phenolphthalein solution test and pH value test.

	Xc/mm	Xc’/mm	Xc/Xc’	Xp/mm
OPC	5.50	14	0.393	8.50
CFA-10	4.35	12	0.363	7.65
CFA-20	3.33	10	0.333	6.67
CFA-30	2.48	10	0.248	7.52
CFA-40	1.97	8	0.246	6.03

## Data Availability

The data presented in this study are available on request from the corresponding author.
